# Knowledge, perception, and attitude toward COVID-19 vaccines in the Ho township, Volta region

**DOI:** 10.3389/fpubh.2025.1516413

**Published:** 2025-07-28

**Authors:** Vivian Tackie, Christiana Asiedu, Ewoenam Darkey, Beatrice Hammond, Linda Jailer, Janet Osei Konadu, Samuel Otieku Cyril, Williams Marie Noel, Isaac Aidoo Erzuah

**Affiliations:** ^1^Department of Public Health Nursing, School of Nursing and Midwifery, University of Health and Allied Health Sciences, Ho, Ghana; ^2^Department of Adult Health, School of Nursing and Midwifery, College of Health and Allied Health Sciences, University of Cape Coast, Cape Coast, Ghana

**Keywords:** Africa, COVID-19 vaccines, cross-sectional, knowledge, practice, public health education

## Abstract

**Introduction:**

Vaccination has emerged as a critical strategy for combating the pandemic and preventing the emergence of new variants. Achieving population-level immunity through vaccination remains essential to reduce disease transmission and protect individuals.

**Objectives:**

This study aimed to assess the knowledge, perceptions, and attitudes toward COVID-19 vaccines.

**Methods:**

A cross-sectional survey was conducted, utilizing simple random and stratified sampling methods to select 443 participants. The response rate was 99.5% (441). A structured questionnaire was used to collect the data. Univariate, bivariate, and multivariate analysis was done. The study was conducted in July, 2022.

**Results:**

Most participants were female, aged 18–65. Over half showed good COVID-19 knowledge, primarily obtained through media and family. Tertiary-educated respondents demonstrated significantly better understanding. While 53.0% believed post-vaccination infection was possible, 56.1% were willing to be vaccinated, though 53.0% would not encourage others. Nearly 70% would refuse a second dose after experiencing reactions to the first. The statistical analysis revealed that COVID-19 knowledge levels were significantly associated with educational attainment (*p* = 0.000), ethnic group (*p* = 0.000), religious affiliation (*p* = 0.015), and employment status (*p* = 0.000), but not with gender (*p* = 0.623) or marital status (*p* = 0.117). The logistic regression model (*p* < 0.00) revealed that tertiary education (AOR = 30.204, *p* < 0.000) and primary education (AOR = 3.466, *p* < 0.047) significantly increased likelihood of good COVID-19 knowledge compared to no education, while Akan ethnicity (AOR = 0.161, *p* < 0.012) was also a significant predictor.

**Conclusion:**

Targeting individuals with lower educational attainment can help bridge the knowledge gap and foster positive attitudes toward COVID-19 vaccines, ultimately contributing to effective virus control and improved public health outcomes. The study underscores the need for educational programs to improve vaccination uptake in Ghana, emphasizing adherence to public health measures.

## Introduction

The first case of COVID-19 was reported in Africa on February 14, 2020. Since then, the region has faced significant challenges in managing the virus’s spread. As of June 27, 2021, the World Health Organization (WHO) had reported over 3.9 million confirmed cases and 94,217 deaths across the African continent (1). In response, governments have implemented various public health measures globally. These include early case detection, extensive testing, screening, contact tracing, health education, physical distancing, and quarantine protocols. Vaccination has emerged as a critical strategy for combating the pandemic and preventing the emergence of new variants (1, 2). Africa has received approximately 672 million doses of COVID-19 vaccines, primarily through the COVAX initiative, bilateral agreements, and the African Union’s Vaccines Acquisition Trust (2). Achieving population-level immunity through vaccination remains essential to reduce disease transmission and protect individuals. Achieving population-level immunity through vaccination is essential for reducing disease transmission and protecting individuals from the virus. However, vaccine acceptance and awareness of COVID-19 are influenced by factors such as knowledge, attitudes, and perceptions (3). These elements significantly impact public acceptance of COVID-19 vaccines.

Previous research highlight the need to address knowledge gaps and attitudes toward preventive measures to enhance vaccine acceptance. In Africa, cultural beliefs, religious diversity, and vaccine hesitancy pose challenges to understanding attitudes regarding COVID-19 and its prevention (4). Studies in Nigeria and Ghana have shown disparities in knowledge distribution among the population (5, 6). While many Ghanaians possess adequate knowledge about COVID-19, hesitancy and disbelief regarding the virus’s existence have also been reported (7). In specific regions, such as Ho Township, concerns have arisen about the potential transmission of diseases from bats to humans. Vaccine hesitancy and disbelief in COVID-19’s existence are particularly noted among Ghanaians, especially in the Volta region. This study investigates the knowledge, perceptions, and attitudes of adults in Ho toward COVID-19. The findings offer valuable evidence to guide public health policy and tailor health education and vaccination programs to effectively address gaps and improve community health outcomes. The findings will also clarify the factors affecting vaccine acceptance and inform strategies to improve vaccination rates in the region.

## Objective of the study

1. To assess the knowledge of COVID-19 among Ghanaians living in Ho Township, Volta Region.
2. To explore the perception about COVID-19 vaccine among Ghanaians living in Ho Township, Volta Region.
3. To determine the attitude of Ghanaians toward COVID-19 vaccine in Ho Township, Volta Region.

## Methods

### Research design

A cross-sectional survey design was employed for the study. Unlike other research designs, the survey is a flexible research design and can elicit both quantitative and qualitative data depending on how it is structured and administered. Hence this design is suitable for the study.

### Research setting

The study was conducted in Ho Township, Volta Region, the administrative capital of the region in Ghana. Located between latitudes 6°20′ N and 6°55′ N and longitudes 0°12′E and 0°53′E, Ho shares borders with Adaklu and Agotime-Ziope District to the south, Ho West District to the north and west, and the Republic of Togo to the east. The population of Ho is 114,472, with a slight majority of females (59,579) compared to males (54,893). Approximately 62% of the population lives in urban areas, and agriculture serves as the primary economic activity. The Ewe ethnic group is predominant, and Christianity is the most widely practiced religion, comprising 91.9% of the population (8). Ho was chosen for its mix of urban and rural settings, cultural uniformity (mainly Ewe and Christian), and agriculture-based economy. As a regional capital, it offers practical access and represents similar mid-sized towns in Ghana. Its location near district and international borders also adds relevance. Results may not reflect all of Ghana due to regional differences. However, findings are valuable for areas with similar demographics and settings. Broader studies can build on this for wider applicability.

### Study population

Population refers to the aggregation of elements from which the sample is selected (9). The population for this study was adults both male and female who have lived in Ho for at least 6 months and are above the age of 18 years.

### Sample and sample size determination

The sample for this study was determined using Cochran’s formula, which is commonly used for calculating sample sizes in populations with unknown finite sizes. This formula ensures that the sample is representative, manageable, and cost-effective for the population being studied.

n= $\frac{z^{2}\mathrm{pq}}{d^{2}}$ Where n = sample size desired.

z = level of confidence adapted from the standard normal distribution (for a level of confidence of 95%, *z* = 1.96, for a level of confidence of 99%, *z* = 2.575).

p = estimated proportion of outcome of interest (*p* = 0.5).

d = tolerated margin of error (0.05).

Therefore, *n* = (1.96)^2^ (0.5) (1–0.5)/(0.05)^2^ *n* = (3.8416) (0.25)/(0.0025) *n* = 384.16.

To account for a potential non-response rate, we add 15.1% to the sample size. This was based on studies on maternal health where the authors used between 15 and 15.2% non-response adjustment factor (10–13).


$$\begin{array}{l} n+(10\%\times n)n+(15.1\%\times 384.16)=n+(0.151\times 384.16)385\\ \times 1.151=443.135=443 \end{array}$$

Therefore, the total sample size for the study is 443 adults living in the Ho for at least 6 months.

### Sampling procedure

According to Turner (14), the sampling method is the selection of a subset of items or entities from a population that the researcher seeks to study. In this study, a combination of simple random sampling and stratified sampling techniques was employed to ensure a representative selection of participants from the population of Ho. The city’s sub-communities served as strata, each assigned a unique identification code to account for geographical variations. To facilitate random selection, a comprehensive list of residents was obtained through official records, community leaders, and local census data; where unavailable, household enumeration was conducted to create a sampling frame. Within each stratum, individuals or households were assigned numbers, and a random number generator was used to select participants. Selected individuals were then approached through in-person visits by trained field researchers, telephone calls if contact details were available, or community announcements and invitation letters distributed with the help of local leaders. This approach ensured a systematic and unbiased selection process, strengthening the validity and reliability of the study.

### Data collection instrument

The research instrument for this study was a structured questionnaire comprising closed-ended and open-ended questions. It was designed to gather precise and concise information from respondents. The questionnaire consisted of four sections: demographic characteristics, knowledge about COVID-19, perception of COVID-19 vaccine, and attitude toward COVID-19 vaccine. Likert scales were used to assess perception and attitude.

### Validity and reliability

To ensure the validity of the research instrument, the questionnaire underwent a rigorous content validation process. Initially, it was reviewed by three experts in Public Health, each with specialized knowledge in disease epidemiology. These experts assessed the instrument for relevance, clarity, and alignment with the study objectives, offering feedback that was incorporated into a revised version. Following this expert review, the questionnaire was pretested in Hohoe, a setting representative of the target population. During this phase, construct validity was examined using Cronbach’s alpha coefficient, which evaluates the internal consistency of the instrument. This process confirmed that the items on the questionnaire effectively measured the intended constructs.

To determine the reliability of the instrument, the test–retest method was employed during the pretest phase in Hohoe. This involved administering the same questionnaire to the same group of respondents at two different points in time under similar conditions. The consistency of responses between the two administrations was assessed using Pearson’s correlation coefficient, yielding a high correlation value (*r* = +0.86), which indicates strong test–retest reliability. Based on the findings from the pretest and feedback received from participants, the questionnaire was revised to improve clarity and relevance. After these modifications, it was subjected to a second expert review to ensure that the revised instrument maintained consistency and accurately captured the intended data over time. These steps collectively ensured the questionnaire’s robustness in terms of both validity and reliability.

### Data collection procedure

Data collection was conducted through face-to-face interviews using paper-based questionnaires, ensuring direct engagement with respondents. Before participation, all respondents were provided with detailed information about the study, including its aim, objectives, privacy protection, and confidentiality. Risks, benefits, and consent procedures were thoroughly explained, and participants had the freedom to withdraw from the study at any time. To maintain data quality, completed questionnaires were reviewed on-site, and clarifications were made where necessary. This systematic approach strengthened the validity and reliability of the study while ensuring ethical research practices. The study was conducted between July and August 2022.

### Data management and analysis

Following data collection, the dataset was cleaned and organized using Microsoft Excel before being imported into SPSS version 22 for statistical analysis. Descriptive statistics (univariate analysis) were performed to summarize demographic characteristics and responses to perception-related items. Knowledge-related variables were assessed using a combination of univariate, bivariate, and multivariate analyses. Responses were scored based on correctness, and both total and average knowledge scores were computed. Bivariate associations between demographic variables and knowledge levels were evaluated using Chi-square tests. Multivariate analysis was conducted using binary logistic regression to identify independent predictors of higher knowledge scores. Perceptions regarding the COVID-19 vaccine were analyzed using descriptive statistics. Attitudinal responses were assessed through measures of central tendency and dispersion, including the mean, median, and standard deviation.

### Ethical consideration

The study adhered to ethical guidelines by obtaining clearance from the University of Health and Allied Sciences Research Ethics Committee (ERC) (UHAS-REC A.11[133] 21–22) and an introductory letter from the Department of Nursing. Hospital Management granted permission, and participants provided informed consent, ensuring voluntary participation and understanding of the study’s purpose and procedures.

## Results

### Demographic characteristics of respondents

Table 1 represents the demographic characteristics of the study respondents. Out of the 441 respondents recruited, the majority were females (273; 61.9%), Christians (272; 61.7%), and minimum age of 18 and maximum age of 65 years, with a mean age of 34.49, SD = 12.10. In terms of marital status, most of the respondents (218; 49.4%) were married, and Ewes were the major ethnic group (128; 29.0%). However, most of the respondents (193; 43.8%) were not employed.

**Table 1:** Demographic characteristics of respondents.

Demographic characteristics	Frequency *N* = 441	Percentages (%)
Age categories (years)		
≤30	197	44.7
31–50	170	38.5
≥51	74	16.8
Gender		
Male	168	38.1
Female	273	61.9
Employment status		
Formally employed	88	20.0
Self employed	160	36.3
Not employed	193	43.8
Religion		
Christianity	272	61.7
Islam	128	29.0
Traditional	41	9.3
Marital status		
Single	151	34.2
Married	218	49.4
Divorced/widowed	31	7.0
Cohabiting	41	9.3
Ethnic background		
Akan	99	22.4
Ewe	128	29.0
Guans	116	26.3
Ga	87	19.7
Others	11	2.5
Educational qualification		
Tertiary	127	28.8
Secondary	170	38.5
Junior secondary	75	17.0
Primary	39	8.8
None	30	6.8

### Sources of information on COVID-19 among respondents

**Figure 1:**
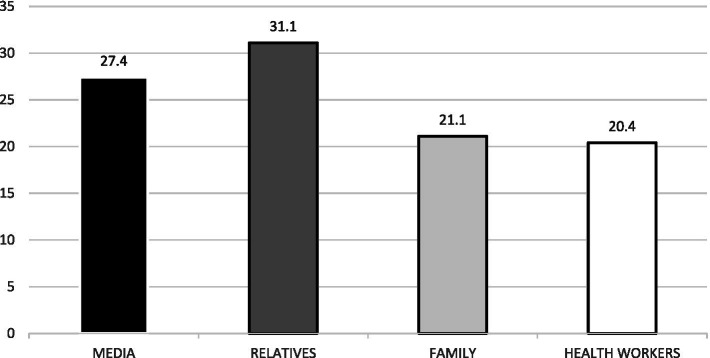
Source of information on COVID-19 disease.

Awareness of COVID-19 among respondents was 100.0%. Figure 1 shows the source of information on COVID-19 disease. Each of the respondents had heard of COVID-19 before. Most of the respondents, 121 (27.4%), heard of it from the media, 137 (31.1%) from relatives, 93 (21.1%) from family, and 90 (20.4%) from health workers.

### Signs and symptoms of COVID-19

**Figure 2:**
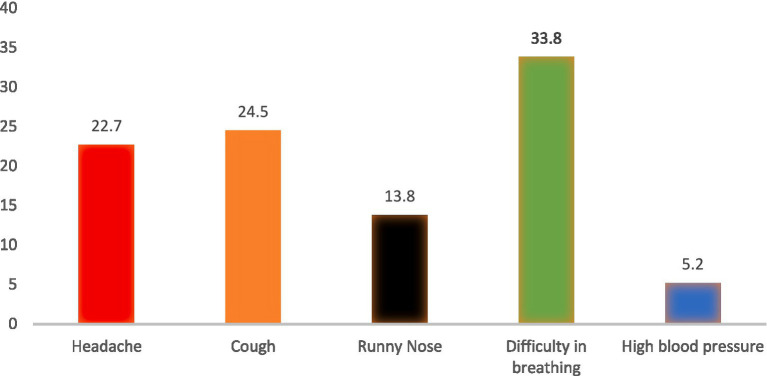
Signs and symptoms of COVID-19 disease.

Figure 2 shows the details of symptoms associated to COVID-19 by respondents. The major COVID-19 symptoms identified were Headache 100 (22.7%), Cough 108 (24.5%), Runny noe 61 (13.8%), High blood pressure 23 (5.2%) and difficulty breathing 149 (33.8%).

### Level of knowledge on COVID-19 among respondents

**Figure 3:**
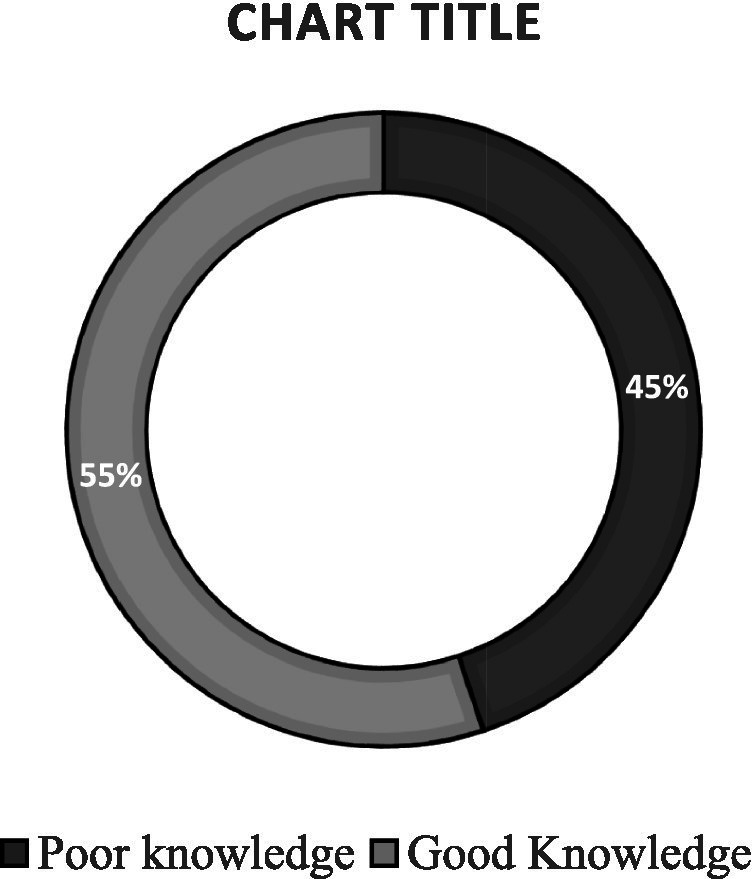
Level of knowledge on COVID-19.

Figure 3 presents the distribution of knowledge scores regarding COVID-19. The knowledge assessment had a maximum achievable score of 14. Among respondents, the observed scores ranged from 1 to 10, with a mean = 5.61, SD = 1.62. For the purpose of analysis, knowledge levels were categorized based on the mean score. Respondents who scored below the mean were classified as having *poor knowledge*, while those who scored above the mean were classified as having *good knowledge*. Using this criterion, 243 respondents (55.25%) were found to have good knowledge of COVID-19.

### Association between demographics on level of knowledge on COVID-19

Table 2 shows association between respondents’ demographics and the level on knowledge on COVID-19. The statistical analysis reveals significant disparities in COVID-19 knowledge levels across different demographic groups, with educational attainment showing the strongest association (*p* = 0.000), where knowledge progressively increased from those with no education (29.3%) to tertiary education (78.2%). Significant differences were also observed across ethnic groups (*p* = 0.000), with Ewe respondents demonstrating the highest knowledge levels (68.2%) and Guans the lowest (41.2%); religious affiliations (*p* = 0.015), where Christians (61.2%) showed greater awareness than followers of Islam (51.6%) or traditional religions (46.8%); and employment status (*p* = 0.000), with formally employed individuals (68.7%) possessing better knowledge than the unemployed (43.6%). Notably, gender and marital status showed no significant association with COVID-19 knowledge, suggesting that targeted educational interventions should prioritize less educated populations, specific ethnic communities, religious groups, and the unemployed to address these knowledge gaps effectively.

**Table 2:** Association between respondents’ demographics and the level of knowledge on COVID-19.

Subgroup	*n* (Good knowledge)	% within subgroup	Chi-square	*p*-value
Ethnic group			29.894	0.000*
Akan	97,154	53.65		
Ewe	154	68.2%		
Guans	68	41.2%		
Ga	101	62.4%		
Others	86	46.5%		
Total	441	57.6%		
Gender			0.257	0.623
Male	252	58.3%		
Female	248	56.9%		
Total	441	57.6%		
Educational level			91.443	0.000*
None	41	29.3%		
Primary	66	39.4%		
Junior secondary	119	52.1%		
Secondary	167	65.3%		
Tertiary	101	78.2%		
Total	441	57.6%		
Religion			8.346	0.015*
Christianity	330	61.2%		
Islam	124	51.6%		
Traditional	47	46.8%		
Total	441	57.6%		
Employment status			28.307	0.000*
Not employed	133	43.6%		
Self-employed	236	58.5%		
Formally employed	134	68.7%		
Total	441	57.6%		
Marital status			5.892	0.117
Single	176	61.4%		
Married	248	57.3%		
Divorced/widowed	52	48.1%		
Cohabiting	25	52.0%		
Total	441	57.6%		

*p-Value is statistically significant.

### Influence of demographics n the level of knowledge on COVID-19 among respondents

Table 3 shows the influence of respondents’ demographics on knowledge of COVID-19 among respondents. Using a logistic regression model [χ^2^(10) =119.790, *p* < 0.00] which was able to classify 78.2% of the cases, it was established that respondents with tertiary education was 30.2 times more likely to have good knowledge on COVID-19 disease than those without any formal education [aOR = 30.204, CI: 8.615–105.890, *p* < 0.000]. Also it was found that respondents with primary school education were 3.466 times more likely to have good knowledge on COVID-19 than those without any formal education [aOR = 3.466, CI: 1.018–11.803, *p* < 0.047]. The model also established that respondents from the Akan ethnic group were 16.1% times more likely to have good knowledge on COVID-19 than those from other ethnic group [aOR = 0.161, CI: 0.039–0.668, *p* < 0.012].

**Table 3:** Influence of demographics n the level of knowledge on COVID-19 among respondents.

Variable	cOR (95% CI); *p*-value	aOR (95% CI); *p*-value
Ethnic group		
Akan	0.525 (0.419–5.508); 0.525	0.249 (0.058–1.068); 0.061
Ewe	0.371 (0.102–1.353); 0.133	0.161 (0.039–0.668); 0.012*
Ga	0.464 (0.127–1.702); 0.247	0.117 (0.027–505); 0.004*
Guan	0.728 (0.202–2.625); 0.628	0.238 (0.058–0.981); 0.047*
Others	Ref	
Educational qualification		
Tertiary	32.286 (11.26–92.53); 0.00*	30.204 (8.615–105.890); 0.000*
Secondary	3.556 (1.383–9.138); 0.008*	3.176 (0.986–10.231); 0.053
Junior high	2.250 (0.818–6.185); 0.116	2.183 (0.629–7.570); 0.219
Primary	3.091 (1.033–9.247); 0.044*	3.466 (1.018–11.803); 0.047*
None	Ref	
Religion		
Christianity	2.144 (1.100–4.177); 0.025*	1.833 (0.694–4.840); 0.222
Islam	1.326 (0.651–2.701); 0.437	0.814 (0.295–2.250); 0.692
Traditional	Ref	
Employment status		
Formally employed	2.170 (1.238–3.802); 0.007*	1.004 (0.493–2.042); 0.992
Self employed	0.495 (0.323–0.757); 0.001*	0.412 (0.522–1.396); 0.009*
Not employed	Ref	

*p-Value is statistically significant.

### Perception of COVID-19 among respondents

**Figure 4:**
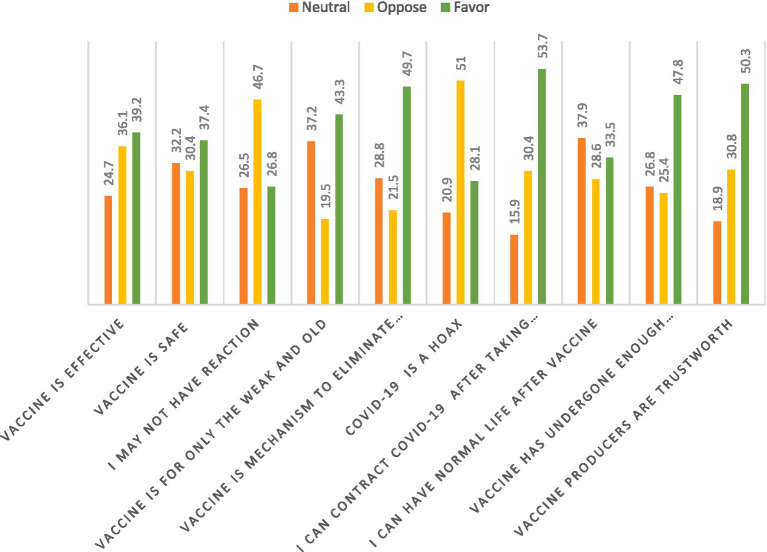
Respondents’ perception on COVID-19 vaccine.

Figure 4 shows the perception respondents had of the COVID-19 vaccine. The COVID-19 vaccine was perceived as effective by 173 (39.2%) of the respondents. With the COVID-19 vaccine being safe for humans, 134 (30.4%) perceived it as unsafe for humans. Also, 206 (46.6%) of the respondents perceived that the vaccine would cause a reaction after taking it. A total of 191 (43.30%) of the respondents perceived the COVID-19 vaccine is for the older adults and the weak. A total of 219 (49.7%) perceived that the COVID-19 vaccine is not a mechanism to eliminate mankind. Also, 225 (51%) indicated that COVID-19 is not a hoax but a vaccine to prevent COVID-19. More than half of the respondents, 237 (53.7%) of the respondents also agreed that they can contract COVID-19 after taking the vaccine. Furthermore, 167 (37.9%) were undecisive as to whether they will have normal life after taken the vaccine or not, and 211 (47.8%) perceived that the vaccine has undergone enough clinical trial so it is safe for usage. Additionally, about half of the respondents, 222 (50.3%), reportedly indicated that vaccine producer are trustworthy.

### Attitude toward COVID-19 vaccine

In terms of attitude toward COVID-19 vaccines, 194 (56.1%) of the respondents reported that they will vaccinate if they had chance. Also, 134 (53.0%) reported that they will not encourage their relatives or friends to vaccinate against the COVID-19. A total of 229 (51.8%) indicated that the vaccination card will not be the reason why they will vaccinate. In terms of route of vaccination, 209 (52.5%) indicated that they will take the vaccine if the vaccine was through oral route. Additionally, 308 (69.7%) reported that they will not take the second shot if they react to the first COVID-19 vaccine shot.

### Level of attitude toward COVID-19 vaccine

**Figure 5:**
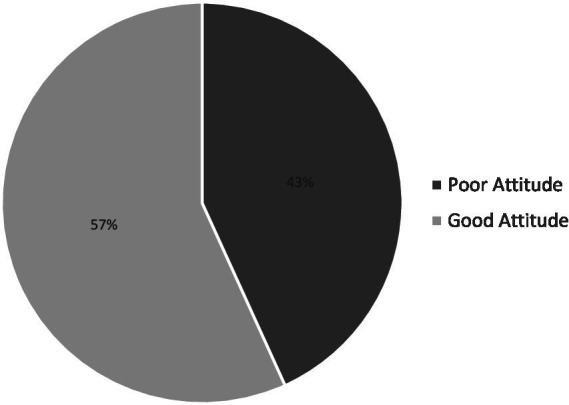
Level of attitude toward COVID-19 vaccine.

By categorizing the attitude response through a summary of the response, it was found that the mean score for the attitude was 11.848, with a median of 12.000 and a standard deviation of 2.887. The maximum score and minimum score were 18.0 and 5.0, respectively. It was found that 56.8% had a good attitude toward the COVID-19 vaccine. Figure 5 shows the category of attitude toward the COVID-19 vaccine.

## Discussion

### Knowledge on COVID-19

Numerous studies conducted across various regions have consistently demonstrated a high level of awareness regarding COVID-19. Mukhlis et al. (15) reported comparable findings, identifying relatives, friends, and media as the primary sources of information, whereas healthcare workers played a less prominent role. Similarly, Almalk et al. (16) and Alali et al. (17) found that media and family members were the main channels through which individuals obtained information about COVID-19. In the present study, 55.25% of respondents exhibited a good level of knowledge about COVID-19, which is in line with findings from similar studies conducted in Ghana and Uganda (18, 19), Educational attainment was identified as a significant determinant of COVID-19 knowledge and awareness, with higher levels of education correlating with increased knowledge (20). Access to media, particularly television, was also found to enhance knowledge acquisition in previous studies, corroborating the results of the current research. Importantly, this study also identified additional factors—such as employment status and ethnic background—as being associated with variations in COVID-19 knowledge. Notably, no conflicting findings were reported in earlier literature. Overall, comprehensive education initiatives and the effective use of media as an informational tool can play a crucial role in enhancing public understanding of COVID-19.

The findings from Ho indicate that just over half (55.25%) of respondents possessed good knowledge of COVID-19, with education emerging as the most influential factor. This trend is consistent with studies across various global regions, underscoring education’s pivotal role in shaping public health awareness. In Asia, for example, a study in China found that individuals with higher education levels were significantly more knowledgeable about COVID-19 prevention and symptoms (21). Similarly, research in India showed that tertiary-educated individuals were more likely to follow correct health protocols and exhibit higher awareness (22). These align closely with the present study, where tertiary-educated respondents were 30 times more likely to possess good knowledge than those without formal education.

In Europe, a study in Italy reported that COVID-19 knowledge was also strongly associated with educational status, particularly in the early stages of the pandemic, influencing adherence to safety guidelines (23). This reflects the Ghanaian context, where knowledge levels scaled with education, from 29.3% among those with no education to 78.2% among those with tertiary education. North America shows similar patterns. In a U. S.-based study, higher education was associated not only with better COVID-19 knowledge but also with greater trust in scientific sources and higher compliance with public health measures (24). Across Africa, regional variations are evident, but educational attainment remains a strong predictor. A study in Nigeria revealed that individuals with higher education levels demonstrated better understanding of transmission routes and prevention (25). Likewise, findings from Ethiopia and Kenya mirrored the trend, showing better knowledge among urban and more educated populations (26).

Ethnicity and religion also played significant roles in the Ho study. Ewe respondents demonstrated the highest knowledge (68.2%), and Christians were more informed (61.2%) than followers of other faiths. Similar socio-cultural influences have been observed elsewhere. In South Africa, disparities in COVID-19 knowledge among ethnic and linguistic groups were linked to historical inequalities in access to information and healthcare (27). Religious affiliation was also a significant variable in a Middle Eastern study, where variations in pandemic awareness were partly attributed to differing interpretations of religious guidance (28). This highlights the importance of culturally tailored communication strategies. Employment status further influenced knowledge, with formally employed individuals being more informed, likely due to workplace sensitization efforts and access to structured communication channels. This pattern echoes findings from studies in Brazil and Pakistan, where employment in formal sectors correlated with better awareness and lower susceptibility to misinformation (29, 30).

Interestingly, the study found no significant gender or marital status differences, contrasting with studies in Bangladesh and the United States, where women were found to be more cautious and informed about COVID-19 risks (48, 49). This might reflect local cultural dynamics in Ho, where access to information may not differ significantly by gender. The logistic regression model adds robustness to these findings. The high adjusted odds ratio (AOR) of 30.2 for tertiary education indicates a very strong influence, while primary education also showed a significant effect (AOR = 3.466). Ethnic differences, though smaller, were still notable (e.g., Akan AOR = 0.161), supporting the need for targeted interventions.

Overall, these findings underscore the vital role of education, structured communication channels, and socio-cultural considerations in shaping public health awareness. Leveraging media and tailoring health communication to diverse population groups can significantly enhance pandemic preparedness and response.

### Perception of COVID-19 vaccines

The findings of this study reveal a complex and concerning landscape of vaccine hesitancy among respondents in Ho, characterized by widespread mistrust, misinformation, and skepticism about the safety and efficacy of COVID-19 vaccines. A significant proportion of participants expressed doubts about the intentions behind vaccine production, with many perceiving the vaccines as tools for population control a belief rooted in conspiracy theories and distrust in global health institutions. These perceptions sharply contrast with findings by Amo-Adjei et al. (31), who reported high levels of trust and willingness to vaccinate among both the general public and religious/traditional leaders in Ghana. This disparity highlights the importance of regional context and the influence of localized beliefs and information sources on vaccine perceptions.

Mistrust in vaccine producers and health authorities, driven by concerns over profit motives, inadequate research, and perceived lack of transparency, has been documented across various studies (32–34). In this study, only 39.1% of respondents believed the vaccine to be effective, while 30.3% deemed it unsafe, and 46.6% expected adverse effects—figures that underscore deep-seated concerns about vaccine reliability and the integrity of the institutions promoting them. These attitudes mirror sentiments expressed in other regions where vaccine rollouts faced public resistance, often fueled by limited health literacy and competing narratives in traditional and digital media. The skepticism observed in Ho is not unique to Ghana. For instance, in Nigeria, Zhong et al. (21) documented widespread vaccine hesitancy, with nearly half of the respondents expressing doubts about safety. Similarly, Osur et al. (35) found that misinformation and a lack of targeted public engagement contributed to hesitancy in Kenya. In India, Roy et al. (22) reported that public trust increased only after prominent figures endorsed vaccination and authorities improved transparency, indicating the power of trusted voices and clear communication in shaping public opinion. European trends have been mixed: while countries like France reported high levels of vaccine hesitancy^36.^ Germany and Sweden saw more positive attitudes, linked to greater trust in government and public health systems (36).

In North America, vaccine skepticism has been particularly notable among minority communities and politically conservative groups, due in part to concerns about the speed of vaccine development and authorization (37). The finding that 47.7% of respondents in Ho believed the vaccine underwent sufficient clinical trials suggests partial trust in scientific processes, yet also signals ongoing uncertainty. Additionally, the belief held by 53.0% of respondents that they could contract COVID-19 even after vaccination reflects persistent misunderstandings about vaccine efficacy, especially regarding breakthrough infections—a global challenge during the early phases of vaccine rollout. Similar misconceptions were seen in Brazil, where contradictory messaging from leadership undermined public confidence (38). Further, the moderate levels of trust in vaccine producers (50.2%) and the fact that 50.9% of participants did not believe the vaccine to be a hoax suggest the presence of an informational divide, where factual knowledge coexists with persistent conspiracy beliefs. The notion that the vaccine is intended primarily for the older adults and vulnerable, believed by 43.0% of respondents, reflects a misinterpretation of risk-based vaccine prioritization—a theme also observed in South Africa and China (27, 39). Moreover, the 28.7% who remained neutral on whether the vaccine was designed to eliminate mankind illustrates the deep-rooted nature of such theories, particularly in contexts with low historical trust in institutions (40).

These findings underscore the urgent need for context-specific health communication strategies. Public health campaigns must not only disseminate factual information but also address the emotional and cultural dimensions of vaccine hesitancy. Community engagement, especially through trusted local figures such as healthcare workers, religious leaders, and educators, is essential. Transparency around clinical trials, side effects, and the development process should be prioritized, along with active debunking of myths using relatable and culturally sensitive narratives. Ultimately, improving vaccine acceptance requires a multi-faceted approach one that combines scientific communication with trust-building efforts across all levels of society.

### Attitude toward COVID-19 vaccine

The current study revealed a complex landscape of attitudes toward COVID-19 vaccination among respondents in Ho. The study revealed that 43.9% of respondents exhibited a negative attitude toward vaccination, with more than half indicating they would not encourage friends or family members to receive the COVID-19 vaccine. In contrast, other studies have reported more favorable attitudes. For instance, Asres and Umeta (41) found that 57.9% of participants held a positive attitude toward vaccination, while Danabal et al. (42) reported a 50.0% rate of positive sentiment among their respondents. Similarly, Kakuru et al. (43), in their study conducted in Uganda, observed that participants with a positive attitude were significantly more likely to get vaccinated, with only a 12% likelihood of vaccine refusal among this group. These variations highlight the importance of understanding the underlying factors contributing to vaccine hesitancy. Such insights are critical for designing effective and targeted vaccination campaigns. It is also important to acknowledge that this study was conducted in a single region, which may limit the generalizability of its findings to other geographical or cultural contexts.

The results reveal a mixed yet slightly positive attitude toward the COVID-19 vaccine among respondents in Ho, with 56.8% demonstrating a good overall attitude. However, deeper insights suggest lingering skepticism and behavioral resistance that could undermine effective vaccine rollout. While 56.1% indicated willingness to vaccinate if given the chance, a contradictory 53.0% stated they would not encourage others (relatives or friends) to get vaccinated. This suggests a gap between personal choice and collective endorsement—a phenomenon commonly observed in vaccine ambivalence, where individuals may accept vaccines personally but hesitate to promote them due to social or cultural pressures (34). Similar patterns have been observed in other regions. For instance, in India, while over 60% expressed willingness to take the vaccine, only about 40% were willing to recommend it to others due to uncertainty about long-term effects (44). Likewise, in South Africa, a study found that although many accepted the personal benefit of vaccination, social media misinformation led to reluctance in advocating for it publicly (27).

A notable 51.8% of respondents in Ho reported that a vaccination card would not influence their decision, indicating that incentives like travel documents or mandates might not significantly improve uptake unless coupled with trust-building. Similar findings were reported in Nigeria, where only 45% believed that the vaccination card (used for travel or work clearance) was a motivator (45). In contrast, in European countries like Italy and France, digital vaccine passes were shown to be effective in encouraging uptake, especially among the hesitant (46). Another key finding was that 52.5% of respondents preferred the vaccine if it was oral, rather than injectable. This aligns with behavioral studies showing that fear of needles and perceived pain significantly reduce vaccine acceptance globally, including in the U. S. and Canada (47). Innovations in oral or nasal vaccines have been suggested as alternatives to boost uptake in such populations.

The most alarming insight, however, is that 69.7% of respondents said they would not take the second dose if they experienced a reaction to the first shot. This points to a major vulnerability in full immunization strategies. In Bangladesh, a study showed similar dropout intentions due to side effects, where 65% of individuals who had mild post-vaccine symptoms expressed unwillingness to complete the dose schedule (40). This underlines the importance of pre-vaccination counseling and post-vaccine follow-up to build confidence. The statistical summary further supports the notion of a relatively moderate attitude level, with a mean attitude score of 11.85 (out of a possible 18), and a standard deviation of ±2.89, indicating variability in sentiment. This variability is echoed across many developing nations, where vaccine attitude is often shaped by education, misinformation, and healthcare experiences.

It is important to acknowledge that this study was conducted in a single region, and the findings may not be universally generalizable. Nonetheless, the insights offer valuable guidance for designing culturally tailored, trust-based interventions aimed at improving vaccine uptake. Again, the study offers critical insights that can directly inform ongoing public health efforts by government and non-governmental organizations (NGOs) in Ghana.

Programs such as the Ghana Health Service’s COVID-19 Vaccination Campaign and initiatives supported by Gavi, the Vaccine Alliance, and UNICEF have been actively working to improve vaccine uptake through community outreach, education, and mobile vaccination units. These efforts often rely on community health workers and local media to disseminate accurate information and counter vaccine misinformation. The findings from Ho particularly the reluctance to advocate for vaccination, low influence of vaccine cards, and fears about side effects highlight areas where these programs can be strengthened. For instance Targeted Education Campaigns, Culturally-Sensitive Messaging, Non-injectable Vaccine Promotion, and Community Advocacy Training. Finally, collaboration between the Ghana Health Service, local NGOs like SEND Ghana and Hope for Future Generations, and international partners can be enhanced by integrating these insights into program design and evaluation. Using data-driven strategies informed by regional research like this study can improve the effectiveness of outreach and ultimately increase vaccination rates.

## Conclusion

The study highlights that patient willingness is a crucial first step in successful vaccination efforts, and enhancing this willingness through targeted education is essential. Findings from Ho reflect global trends, showing that factors such as education, ethnicity, religion, and employment significantly influence COVID-19 knowledge and attitudes. Despite a moderate level of vaccine acceptance, concerns about safety, mistrust in authorities, and reluctance to recommend vaccination to others remain key barriers. These insights emphasize the need for context-specific, community-led public health campaigns—especially through mass media and bulk messaging—to combat misinformation and build trust. While the findings provide valuable direction, further research in diverse settings is needed to inform nationwide strategies and ensure broader vaccine uptake.

### Limitations of the study

1. The study was conducted solely in Ho Township, in the Volta Region of Ghana. This localized scope limits the generalizability of findings to the broader Ghanaian population or other regions with different socio-cultural and economic dynamics.
2. As a cross-sectional study, data were collected at a single point in time. This design limits the ability to assess changes in knowledge, attitudes, or perceptions over time or in response to evolving information about COVID-19 and vaccination campaigns.
3. The study focused primarily on quantitative measures. Qualitative methods such as interviews or focus group discussions could have enriched the findings by exploring the reasons behind vaccine hesitancy or misinformation more deeply.
4. One notable limitation of this study is the wide confidence interval (CI) associated with the adjusted odds ratio (AOR = 30.204, CI: 8.615–105.890). While the effect size appears strong and statistically significant, the breadth of the confidence interval suggests a high degree of variability.

## Data Availability

The raw data supporting the conclusions of this article will be made available by the authors, without undue reservation.
